# Anaplastic Large Cell Lymphoma Associated with Breast Implant: A Case Report

**Published:** 2012-01

**Authors:** Afsaneh Rajabiani, Hosein Arab, Abolhasan Emami, Ali Manafi, Navid Bazzaz, Hiva Saffar

**Affiliations:** 1 Department of Pathology, Tehran University of Medical Sciences, Tehran, Iran.; 2 Plastic surgeon, Parsian Hospital, Tehran, Iran.; 3 Department of Plastic and Reconstructive Surgery, Tehran University of Medical Sciences, Tehran, Iran.

**Keywords:** Anaplastic Large Cell Lymphoma, Anaplastic Lymphoma Kinase, Silicone breast implant

## Abstract

Primary breast lymphoma represents less than 1% of all primary breast malignancies and most primary breast lymphomas are of B-Cell origin. The association of anaplastic lymphoma kinase (ALK) negative anaplastic large cell lymphoma (ALCL), a very rare form of primary breast lymphoma, with silicone-filled breast implants has been suggested and several case reports supported this proposal, especially in Western countries. Here we describe one of the first cases of primary breast ALK-negative ALCL in association with saline-filled silicone breast implants evaluated in Iran, where the rising number of breast reconstructive and aesthetic surgeries would commit both surgical pathologists and plastic surgeons to be familiar with this entity.

## INTRODUCTION

Silicone implants are used for augmentation of breast for cosmetic purposes or after lumpectomy, or mastectomy due to breast malignancies.^1^ Silicone products have proven to be capable of eliciting inflammatory and fibro-proliferative responses^2^ or proposed to contribute in connective tissue disorders.^3^

Primary breast lymphoma is uncommon and constitutes less than 1% of all primary breast malignancies.^4^^-^^6^ Most of primary breast lymphomas are of B-cell origin^6^^-^^8^ and T-cell lymphomas comprise just less than 10% of all non Hodgkin lymphomas (NHL) primarily involving breast.^4^^-^^6^^,^^9^^,^^10^ For the first time in 1995, Duvic *et al.*^11^ suggested that there might be an association between silicone implants and primary breast NHLs.

Anaplastic lymphoma kinase (ALK)-negative anaplastic large cell lymphoma (ALCL) is a rare peripheral T-cell lymphoma.^6^ The first cases of ALCL in association with a saline-filled breast lymphoma implant were reported before.^6^^,^^9^^,^^12^ However, there are limited reports of such association in eastern countries.

Due to improvements in breast cancer managements and life expectancy in developing countries, the short and long term effects of breast reconstructive surgeries are becoming more challenging. Also in step with the rise in life standard levels in such countries, we face more aesthetic breast surgeries. Here we describe one of the first cases of primary breast AlK-Negative ALCL in association with saline-filled silicone breast implant evaluated in Iran.

## CASE REPORT

A 44-year old female patient presented with a clinical history of breast cosmetic augmentation surgery by saline-filled silicone implant, about 14 years ago. She had recently experienced pain, tenderness, breast enlargement and discomfort associated with rather firm indurations around the implant of the right breast, radiating into the right arm, for few months. There was history of radiation of pain into the right arm.

The Sonography was non-diagnostic because of the presence of breast implant. On systemic examination, neither lymphadenopathy nor hepatosplenomegaly were detected. There was no evidence of cutaneous involvement. Imaging studies also ruled out the possibility of any systemic disease.

During the operation, large amounts of unusual fibrinous material were found around the saline-filled breast implant, mainly located in the lower inner quadrant LIQ. Then the suspicious tissues were biopsied and submitted for histological examination. The necrotic debris and surrounding tissue were fixed in 10% buffered formalin. Representative sections of the whole specimen were embedded in paraffin blocks, processed and stained with hematoxylin and eosin (H&E) for routine histological examination; also serial sections for immunohistochemical studies were prepared. Immunohistochemical staining was as performed by using antibodies, summarized in Table 1.

**Table 1:** Antibodies used for immunohistochemical studies

**Target protein**	**Clone**	**Source**
LCA^1^	2B11+PD7126	DAKO
CD3	polyclonal	DAKO
CD5	CD5/54/F6	DAKO
CD7	CBC.37	DAKO
CD20	L26	DAKO
CD30	Ber-H2	DAKO
EMA^2^	E29	DAKO
ALK^3^	ALK1	DAKO
Granzyme B	GrB-7	DAKO
CK	5D3 and LP34	Novocastra

1 LCA: Leukocyte Common Antigen,

2 EMA: Epithelial Membrane Antigen,

3 ALK: Anaplastic Lymphoma Kinase

Under light microscopy, sections revealed predominance of necrosis intermingled with particles of neoplastic tissue of lymphoid origin, composed of many large anaplastic lymphoid cells with large lobulated nuclei, showing rather irregular nuclear membrane and one or more prominent basophilic nucleoli. The cytoplasm was slightly basophilic with prominent Golgi region. Mitotic figures were frequent. There were also many histiocytes, containing abundant pale cytoplasms. Some sections included portions of a dense fibrous capsule, associated with diffuse marked infiltration of eosinophils.

**Fig. 1:**
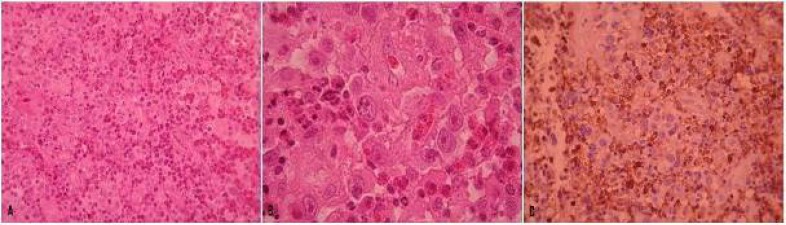
Large anaplastic cells with prominent nucleoli admixed with histiocytes and many eosinophils (A&B) show diffuse positive immunoreaction to Leukocyte common Antigen (LCA) (C).

**Fig. 2:**
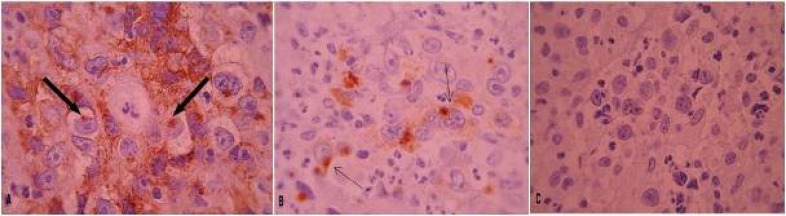
Positive immunoreactivity of lymphoma cells to CD30 (A) and Granzyme B (B) both with paranuclear pattern (arrows) and negative immunoreactivity to AlK (C).

The anaplastic cells exhibited positive immunoreactions for leukocyte common antigen (LCA), CD3, CD30 (Figure 1A) and granzyme B (Figure 1B). However, they were negative for AlK (Figure 1C), epithelial membrane antigen, cytokeratin, CD 5, CD 7 and CD20. The final diagnosis was ALK-negative ALCL (Figure 2A-C).

## DISCUSSION

The silicone-based implants were first marketed in 1962 in United States of America, mainly for augmentation of breast either for cosmetic purposes or after lumpectomy or mastectomy due to breast malignancies.^1^ For decades, the safety of this procedure has been debated.^6^ Although several large epidemiological studies showed neither increased risk of developing malignancies in breast after breast implants^6^^,^^13^^-^^16^ nor increase in rate of well-defined connective tissue disease,^17^ several studies showed silicone breast implants associated with T-cell mediated autoimmune reactions or breast malignancies, especially lymphoma.^2^^,^^6^^,^^8^^,^^9^^,^^12^^,^^18^^-^^25^

Although ALK-negative ALCL is a rare peripheral T-cell lymphoma,^6^ the association of this special type of lymphoma with breast implants is more than that it could be simply explained by chance. Silicones were shown to stimulate T-cell mediated reactions by production of auto anti-bodies.^6^^,^^11^ Since chronic inflammation can play role in development of malignant lymphoma such as *H. Pylori* in gastric lymphoma, it could be possible that exaggerated inflammatory reaction, mediated by silicone implant, is responsible for developing ALCL under such circumstances.^6^

According to Li and Lee study,^6^ unlike the primary breast lymphoma which typically presents as a mass lesion, most patients with implant-associated primary ALCL show implant-related symptoms with or without mass lesions. Seroma formation is the most common presentation of implant related ALCL. Roden *et al.*^8^ called it as seroma associated primary ALCL but Thompson *et al.*^7^ preferred the term as effusion associated-Anaplastic Large Cell Lymphoma (ea-ALCL) for this special situation, because the fluid was a malignant effusion rather than seroma. Our reported case also presented with tenderness at implant site but at surgery peri-implant area was filled by fibrinous material containing cell debris. As Thompson *et al.* reported,^7^ our case also showed no evidence of tissue invasion and malignant cells suspended in the fluid adjacent to implant capsule.

Immunohistochemically, primary breast ALCL cells were strongly positive for CD30 and mostly positive for EMA. LCA and at least one of T-cell markers in association with cytotoxic markers like TIA_1_ or Granzyme B could be helpful in distinguishing this lesion from other differential diagnoses. ALK is mostly negative in silicone-associated cases.^6^ Except for absence of EMA reaction; our case followed the usual pattern of immunohistochemistery.

While no convincing epidemiologic study has proved the association between ALK-negative ALCL and silicone implants, the rising number of case reports has questioned the long term safety of breast augmentation or reconstructive surgeries using silicone implants. By reviewing the literature, as Thompson *et al.*^7^ mentioned, ea-ALCL seems to be a separate category which would not fit the criteria of systemic ALCL in WHO classification. So it must be emphasized that all tissue samples removed due to implant related complications must be carefully examined by pathologists, and all pathologists should be aware of this entity, because it could be easily misdiagnosed on histological evaluation.
